# Poor outcome despite modern treatments: A retrospective study of 99 patients with primary and secondary plasma cell leukemia

**DOI:** 10.1002/cam4.70192

**Published:** 2024-09-03

**Authors:** Camille Tessier, Richard LeBlanc, Jean Roy, Sabrina Trudel, Julie Côté, Marc Lalancette, Jean‐Samuel Boudreault, Émilie Lemieux‐Blanchard, Rayan Kaedbey, Michel Pavic

**Affiliations:** ^1^ Université de Sherbrooke Sherbrooke Quebec Canada; ^2^ Hôpital Maisonneuve‐Rosemont Montreal Quebec Canada; ^3^ Hôpital Charles‐Le Moyne Greenfield Park Quebec Canada; ^4^ Centre Hospitalier Universitaire de Québec (CHUQ), Hôpital de l'Enfant‐Jésus Quebec Quebec Canada; ^5^ Centre Hospitalier Universitaire de Québec (CHUQ), Hôtel‐Dieu de Québec Quebec Quebec Canada; ^6^ Hôpital du Sacré‐Cœur de Montréal Montreal Quebec Canada; ^7^ Centre Hospitalier de l'Université de Montréal (CHUM) Montreal Quebec Canada; ^8^ Jewish General Hospital Montreal Quebec Canada; ^9^ Centre Hospitalier Universitaire de Sherbrooke (CHUS) Sherbrooke Quebec Canada; ^10^ Institut de Recherche sur le Cancer de l'Université de Sherbrooke (IRCUS) Sherbrooke Quebec Canada

**Keywords:** hematologic malignancies, multiple myeloma, plasma cell disorder, plasma cell leukemia, retrospective study

## Abstract

**Background:**

Plasma cell leukemia (PCL) is a rare monoclonal gammopathy, associated with short survival. Because of its very low incidence, only a few cohorts have been reported and thus, information on this disease is scarce. The goal of this study was to better understand the clinical features, prognostic factors, and efficacy of modern treatments in both primary PCL (pPCL) and secondary PCL (sPCL).

**Methods:**

We performed a retrospective, multicenter study of patients diagnosed with PCL, defined as circulating plasma cells ≥20% of total leukocytes and/or ≥2 × 10^9^/L.

**Results:**

We identified 99 eligible PCL patients, of whom 33 were pPCL and 66 were sPCL. The median progression‐free survival (PFS) to frontline treatment and overall survival (OS) were, respectively, 4.8 (95% CI, 0.4–9.2) and 18.3 months (95% CI, 0.0–39.0) for pPCL and 0.8 (95% CI, 0.5–1.1) and 1.2 months (95% CI, 0.9–1.5) for sPCL (both *p* < 0.001). We observed no improvement in OS over time (2005–2012 vs. 2013–2020, *p* = 0.629 for pPCL and *p* = 0.329 for sPCL). Finally, our data suggested that sPCL originates from a high‐risk multiple myeloma (MM) population with a short OS (median 30.2 months), early relapse after stem cell transplant (median 11.9 months) and a high proportion of patients with multiple cytogenetic abnormalities (36% with ≥2 abnormalities).

**Conclusions:**

This study is one of the largest PCL cohorts reported. We are also the first to investigate characteristics of MM before its transformation into sPCL and demonstrate that high‐risk biologic features already present at the time of MM diagnosis. Moreover, our data highlights the lack of improvement in PCL survival in recent years and the urgent need for better treatment options.

## Graphical abstract

In this multicenter study of 99 patients with plasma cell leukemia, we show no improvement in survival over a 15‐year period, despite major advances in the treatment of plasma cell dyscrasias. We are also the first to investigate the clinical characteristics of secondary PCL before leukemic transformation and demonstrate that high‐risk biological features are already present at the time of multiple myeloma diagnosis.

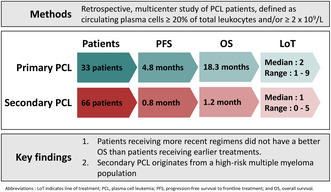

## 1 INTRODUCTION

Plasma cell leukemia (PCL) is a highly aggressive monoclonal gammopathy characterized by plasma cells circulating in the peripheral blood. It is usually divided into primary PCL (pPCL), when occurring de novo, or secondary PCL (sPCL), when a patient previously diagnosed with multiple myeloma (MM) undergoes leukemic transformation. Although first described over a century ago,^1^ the diagnostic criteria for PCL were originally established by Kyle et al., in 1974.^2^ In this seminal paper, PCL was defined by the presence of circulating plasma cells exceeding 2 × 10^9^/L and 20% of total white blood cells (WBC). However, these criteria were deemed too restrictive by many experts and, in 2013, the International Myeloma Working Group (IMWG) determined that either one of these criteria was sufficient for PCL diagnosis.^3^ In late 2021, the diagnostic criteria for PCL were once again updated by the IMWG to include patients with ≥5% circulating plasma cells,^4^ following recent studies demonstrating that this lower threshold results in similar clinical outcomes.^5^, ^6^, ^7^

To establish a diagnosis of PCL, it is currently recommended to assess circulating plasma cells by conventional microscopy.^4^ Flow cytometry appears to be a good candidate to eventually replace manual differential count, as it is more accurate, can confirm clonality and predicts poor prognosis.^8^, ^9^, ^10^, ^11^ However, flow cytometry precision varies greatly across instruments and further validation is required before being widely implemented.^4^, ^12^ Another promising tool for PCL diagnosis is transcriptome analysis. Recent studies have reported that MM and PCL have distinct gene expression profile and thus, transcriptome could be used to diagnose and stratify PCL.^12^, ^13^, ^14^ As for flow cytometry, additional work is nevertheless needed before this newer approach can be applied outside of research protocols.

There is no agreed standard treatment for PCL. Until recently, both pPCL and sPCL patients were systematically excluded from prospective clinical trials and thus, treatments were extrapolated from MM regimens. In the last years, however, a few non‐randomized studies dedicated to PCL have been reported^15^, ^16^, ^17^ and PCL patients are gradually included in high‐risk MM trials.^18^, ^19^, ^20^ Based on these and other retrospective studies, many experts recommend the use of a proteasome inhibitor and autologous stem cell transplant (ASCT) in eligible patients, as these treatments appear to be associated with improved survival.^21^, ^22^, ^23^, ^24^, ^25^, ^26^ For example, in the recent EMN12/HOVON‐129 trial,^16^ the use of carfilzomib, lenalidomide and dexamethasone (KRd) as an induction regimen followed ASCT resulted in favorable survival compared to historical data. Nevertheless, patients included in this study, as in other clinical trials, were highly selected, and real‐life data regarding response to modern treatments are lacking. Therefore, the first aim of this study was to better evaluate the impact of newer therapies, usually studied in MM, on PCL survival in a general population.

Although studies consistently report a significantly poorer prognosis for PCL patients when compared to MM patients,^21^, ^24^, ^26^, ^27^, ^28^, ^29^, ^30^, ^31^ the causes are not fully understood. Few studies have focused on the clinical characteristics specific to PCL, therefore limiting our ability to better target treatments. With its overall survival (OS) of only a few months,^22^, ^30^, ^31^, ^32^ sPCL is often described as a late‐stage complication of heavily pretreated MM. However, it is currently unknown whether this disease originates from an already high‐risk MM or is the result of long‐term selection of an aggressive clone. To our knowledge, no research group has explored the characteristics of patients before their transformation to sPCL. For pPCL, a few research groups have noticed that some patients live significantly longer than expected,^14^, ^24^ but the reasons remain largely unknown. Our second goal was therefore to better understand the specificities of PCL by assessing the impact of various prognostic factors on survival and describing patients who eventually progress into sPCL at the time of MM diagnosis.

## 2 METHODS

### 2.1 Patients

We performed a retrospective, multicenter study of PCL patients diagnosed between January 2005 and December 2020 in eight academic centers in the Province of Québec, Canada. Patients were identified through electronic medical records. Inclusion criteria were circulating plasma cells ≥20% of total WBC and/or plasma cells count ≥2 × 10^9^/L. Patients were excluded if they received treatment for another malignancy after a diagnosis of PCL. The datalock date was July 1, 2022. The study was conducted according to the guidelines of the Declaration of Helsinki, and approval of this study was obtained from the *Comité d'Éthique de la Recherche* (research Ethics Boards) of the CHUM, the CHUQ, the CHUS, the Charles‐Le Moyne Hospital, the Jewish General Hospital, the Maisonneuve‐Rosemont Hospital and the Sacré‐Cœur de Montréal Hospital (multicentric approval number: MP‐31‐2022‐452).

### 2.2 Techniques

Plasma cells percentage was calculated using conventional microscopy of peripheral blood smears, bone marrow aspirates, and bone marrow biopsy. Cytogenetics aberrations were tested by fluorescent in situ hybridization (FISH), with probes varying between institutions and throughout time. The number of patients tested for each cytogenetic abnormality is mentioned in the results and supplementary material sections. Immunophenotype was determined by flow cytometry of circulating or medullary plasma cells. CD38 and CD138 were consistently tested, but other markers (CD56, CD19, CD20, CD117, CD45) varied between institutions and throughout time.

### 2.3 Statistical analysis

Statistical analysis was done using IBM SPSS Statistics 28.0 and Excel 2016. Progression‐free survival (PFS) was defined as the time from diagnosis until progression or death whichever comes first. OS was defined as the time from diagnosis until death from any cause. OS and PFS were estimated by the Kaplan–Meier method and compared by the log rank test. Median follow‐up was calculated using the reverse Kaplan–Meier method. Cox regression univariable and multivariable analyzes were used to assess the impact of various prognostic factors on OS and time to progression, the results of which are reported as hazard ratios (HRs) with 95% of confidence intervals (95% CI). Differences between subgroups were compared by using either the Chi‐square or Fisher exact statistical test. The results were considered significant if the *p* value was <0.05.

## 3 RESULTS

### 3.1 Population and patient characteristics

We identified 125 patients with a diagnosis of PCL (1.4% of our MM population), of whom 99 fulfilled our study criteria. These included 33 patients with pPCL and 66 with sPCL. Of the 26 excluded patients (10 pPCL and 16 sPCL), one was rejected due to concomitant malignancy while all others were excluded due to missing data (mainly elderly patients who declined investigations or patients whose main follow‐up was in another institution).

Baseline characteristics of pPCL and sPCL patients are shown in Table 1. For sPCL patients, Table 1 also describes their clinical characteristics at the time of MM diagnosis. Among pPCL and sPCL, 63.6% of patients met both relative and absolute diagnostic criteria (circulating plasma cells ≥20% and ≥2 × 10^9^/L), 32.3% met only the relative criteria and 4.0% met only the absolute criteria. Circulating plasma cells were found in the peripheral blood of 11 patients with sPCL, at the time of their MM diagnosis. According to the most recent diagnostic criteria, six of these patients would now have been diagnosed with primary plasma cell leukemia (5%–19% of circulating plasma cells) and five would still be considered MM (1%–4% of circulating plasma cells).

**TABLE 1:** Patient characteristics at diagnosis of multiple myeloma (MM), secondary plasma cell leukemia (sPCL) or primary plasma cell leukemia (pPCL). All patients included in the MM category correspond to patients who eventually progressed to sPCL.

Parameters	MM (*n* = 66)	sPCL (*n* = 66)	pPCL (*n* = 33)
Age at diagnosis, years, median (range)	61.8 (35.7–83.4)	64.2 (37.8–85.3)	59.5 (40.7–86.3)
Male sex, *n* (%)	32 (48.5)		19 (57.6)
Plasma cells, median (range)			
Circulating (%)	0 (0–15)	30 (17–76)	30 (17–76)
Circulating (absolute)	0.0 (0.0–1.2)	2.4 (0.3–81.5)	6.3 (1.1–72.8)
Medullary (%)	46 (10–100)	80 (14–100)	90 (50–100)
ISS stage, *n* (%)			
I	11/46 (23.9)	4/31 (12.9)	3/27 (11.1)
II	12/46 (26.1)	6/31 (19.4)	7/27 (25.9)
III	23/46 (50.0)	21/31 (67.7)	17/27 (63.0)
R‐ISS stage, *n* (%)			
I	4/27 (14.8)	0/25 (0)	2/23 (8.7)
II	16/27 (59.3)	10/25 (40.0)	9/23 (39.1)
III	7/27 (25.9)	15/25 (60.0)	12/23 (52.2)
Paraprotein isotype, *n* (%)			
IgG	29 (43.9)	29 (43.9)	10 (30.3)
IgA	19 (28.8)	18 (27.3)	3 (9.1)
IgM	0 (0)	0 (0)	1 (3.0)
Light chain only	18 (27.3)	19 (28.8)	19 (57.6)
Light chain isotype, *n* (%)			
Kappa	34 (51.5)	34 (51.5)	21 (63.6)
Lambda	32 (48.5)	32 (48.5)	12 (36.4)
Biclonal gammopathy, *n* (%)	5 (8.1)	10/62 (16.1)	3/30 (10.0)
CRAB features, *n* (%)			
Hypercalcemia	22 (34.4)	30 (45.5)	22 (66.7)
Renal failure	25 (38.5)	37 (56.1)	23 (69.7)
Hemoglobin <100 g/L	24 (36.4)	56 (84.8)	30 (90.9)
Bone lesions	42/64 (65.6)	25/39 (64.1)	21/32 (65.6)
Other clinical features, *n* (%)			
Platelets <100 × 10^9^/L	6 (9.1)	54 (81.8)	16 (48.5)
Total WBC > 10 × 10^9^/L	7 (10.6)	22 (33.3)	27 (81.8)
Elevated LDH	14/52 (26.9)	46/63 (73.0)	20/30 (66.7)
Elevated β_2_‐microglobulin	35/51 (68.6)	30/31 (96.8)	23/27 (85.2)
Positive Bence Jones	39/52 (79.0)	20/25 (80.0)	13/15 (86.7)
Immunoparesis	52/56 (92.9)	53/55 (96.4)	27/30 (90.0)
Splenomegaly	4/35 (11.4)	12/25 (48.0)	11/27 (40.7)
Cytogenetic abnormalities, *n* (%)			
Normal FISH	10/25 (40.0)	0/13 (0)	2/24 (8.3)
Standard risk abnormalities			
Trisomy	5/25 (20.0)	3/13 (23.1)	5/24 (20.8)
t (11;14)	0/6 (0)	2/4 (50.0)	4/7 (57.1)
High‐risk abnormalities			
t(4;14)	3/17 (17.6)	2/9 (22.2)	2/21 (9.5)
t(14;16)	2/11 (18.2)	2/7 (28.6)	2/12 (16.7)
Del17p	3/21 (14.3)	3/11 (27.3)	5/21 (23.8)
Gain 1q	0/3 (0)	4/6 (66.7)	10/13 (76.9)
Del1p	2/4 (50.0)	2/6 (33.3)	4/8 (50.0)
≥2 abnormalities	9/25 (36.0)	9/13 (69.3)	14/24 (58.3)
≥3 abnormalities	5/25 (20.0)	5/13 (38.5)	9/24 (37.5)
Immunophenotype, *n* (%)			
CD56+	11/14 (78.6)	12/21 (57.1)	9/21 (42.9)
CD19 and/or CD20+	4/21 (19.0)	3/23 (13.0)	7/22 (31.8)

Abbreviations: BM, bone marrow; FISH, fluorescent in situ hybridization; ISS, international staging system; LDH, lactate dehydrogenase; R‐ISS, revised ISS; WBC, white blood cells.

### 3.2 MM characteristic and treatments before sPCL

For the 66 sPCL patients, the median time between initial MM diagnosis and leukemic progression was 27.3 months (interquartile range [IQR], 12.7–41.6). Prior to MM diagnosis, seven patients had also been diagnosed with monoclonal gammopathy of undetermined significance (MGUS) and seven patients with smoldering multiple myeloma (SMM). The median time to progression from MGUS to PCL and from SMM to PCL were 61.9 months (IQR, 50.6–90.6) and 21.2 months (IQR, 11.0–32.3), respectively.

The median number of lines of treatment prior to transformation was 2 (range 1–7). Overall, 28 patients (42.4%) underwent single ASCT and 4 (6.1%) received tandem ASCT‐allogeneic stem cell transplantation (ASCT‐alloSCT). The median time to relapse after SCT was 11.9 months (IQR, 10.3–19.0). When analyzed by year of MM diagnosis (< 2013 vs. ≥2013), we observed no difference in the median time to relapse after SCT (respectively, 12.1 vs. 10.4 months, *p* = 0.348). In our cohort, single ASCT or tandem ASCT‐alloSCT did not result in significantly longer time to PCL progression when compared to those who received chemotherapy alone (31.6 vs. 22.9 months, *p* = 0.164). The treatments administered were highly heterogeneous (Table S1) therefore limiting our ability to assess their individual impact on progression or survival.

### 3.3 PCL treatments

**FIGURE 1:**
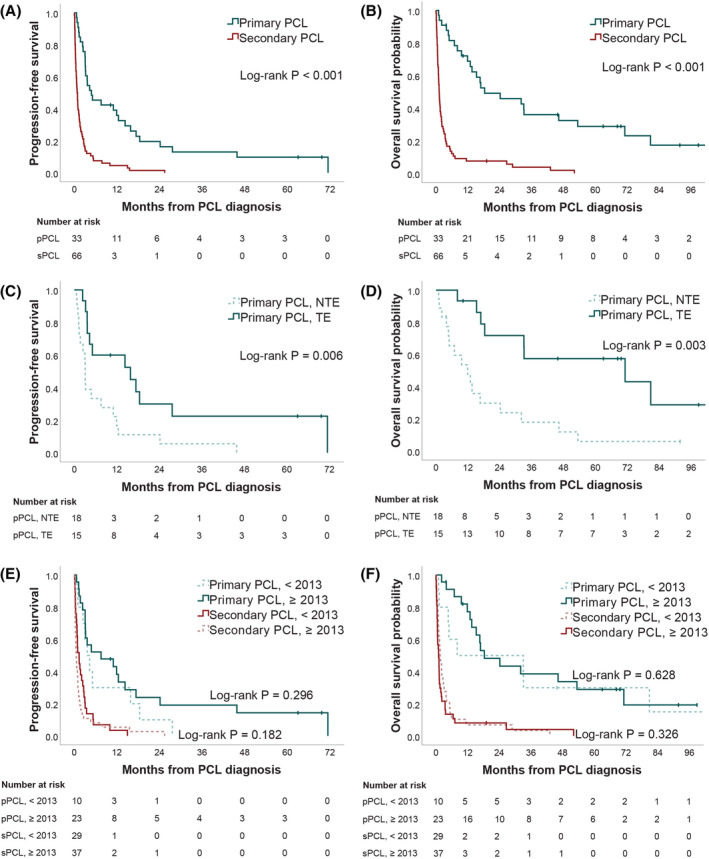
Kaplan–Meier curves for progression‐free survival (PFS) and overall survival (OS) in plasma cell leukemia (PCL) patients. (A) The median estimated PFS was 4.8 months (95% CI, 0.4–9.2) for primary PCL (pPCL) and 0.8 month (95% CI, 0.5–1.1) for secondary PCL (sPCL) (*p* < 0.001). (B) The median estimated OS was 18.3 months (95% CI, 0.0–39.0) for pPCL and 1.2 month (95% CI, 0.9–1.5) for sPCL (*p* < 0.001). (C) The median estimated PFS was 15.8 months (95% CI, 0.0–34.3) for transplant‐eligible (TE) pPCL and 3.0 months (95% CI, 2.9–3.2) for non‐transplant‐eligible (NTE) pPCL (*p* = 0.006). (D) The median estimated OS was 71.1 months (95% CI, 0.0–153.6) for transplant‐eligible (TE) pPCL and 11.9 months (95% CI, 3.9–19.8) for non‐transplant‐eligible (NTE) pPCL (*p* = 0.003). (E) The median estimated PFS was 3.6 months (95% CI, 1.6–5.7) for pPCL diagnosed <2013 and 7.5 months (95% CI, 0.0–18.7) for pPCL diagnosed ≥2013 (*p* = 0.296). The median estimated PFS was 1.3 month (95% CI, 0.5–2.2) for sPCL diagnosed <2013 and 0.5 month (95% CI, 0.3–0.7) for sPCL diagnosed ≥2013 (*p* = 0.182). (F) The median OS was 8.0 months (95% CI, 0.0–51.6) for pPCL diagnosed <2013 and 18.3 months (95% CI, 7.1–29.5) for pPCL diagnosed ≥2013 (*p* = 0.628). The OS was 1.6 month (95% CI, 1.0–2.2) for sPCL diagnosed <2013 and 1.0 month (95% CI, 0.4–1.6) for sPCL diagnosed ≥2013 (*p* = 0.326).

The treatment plan, including decision to proceed to transplantation, was based on physician discretion and patient preference. The median number of treatment lines was 2 (range 1–9) for pPCL and 1 (range 0–5) for sPCL. Among pPCL patients, 30% (10/33) underwent single ASCT, 9% (3/33) underwent tandem ASCT and 6% (2/33) underwent tandem ASCT‐alloSCT. Patients with pPCL who underwent any type of SCT had a significant longer PFS (HR 0.35, 95% CI 0.16–0.76, *p* = 0.008) and OS (HR 0.27, 95% CI 0.11–0.64, *p* = 0.003) than patients treated with chemotherapy alone (Figure 1C,D). Both SCT and chemotherapy subgroups were of similar age at diagnosis (respectively, 59.3 and 61.3 years) and had a comparable proportion of renal failure (respectively, 60.0 and 77.8%, *p* = 0.448). The impact of any type of SCT on sPCL survival could not be assessed, as only two of the 66 sPCL patients received this type of treatment.

As for patients with MM before transformation into sPCL, the detailed analysis of different treatment protocols was not possible due to the great variability of the regimens used (Table S1). From 2005 to 2012, the most frequent frontline treatment was thalidomide with dexamethasone. From 2013 to 2020, the most frequent frontline treatments were bortezomib, doxorubicin, and dexamethasone (PAD), cyclophosphamide, bortezomib, and dexamethasone (CyBorD) or a combination of both (alternating PAD/VCD). As illustrated in Table 2 and Figure 1, more recent regimens (2013–2020) did not improve OS when compared to earlier treatments (2005–2012). Clinical characteristics divided by year of diagnosis are presented in Table S2.

**TABLE 2:** Univariable analyzes for overall survival (OS).

Variable	Primary PCL		Secondary PCL	
	HR (95% CI)	*p* value	HR (95% CI)	*p* value
Demographics				
Male sex	1.41 (0.62–3.24)	0.415	0.71 (0.43–1.19)	0.197
Age ≥ 60 years	1.25 (0.56–2.80)	0.586	1.21 (0.72–2.03)	0.470
Diagnosis after 2012	0.81 (0.34–1.91)	0.629	1.28 (0.78–2.10)	0.329
ISS stage^a^				
II	2.25 (0.44–11.45)	0.327	1.58 (0.44–5.70)	0.482
III	1.12 (0.24–5.14)	0.886	1.35 (0.45–4.01)	0.594
R‐ISS stage^a^				
II	1.21 (0.24–5.98)	0.818	‐–	‐–
III	0.73 (0.15–3.56)	0.698	1.28 (0.55–2.99)	0.563
Paraprotein isotype^b^				
IgA	1.75 (0.35–8.77)	0.497	1.51 (0.82–2.79)	0.186
Light chain	1.02 (0.40–2.61)	0.967	1.37 (0.76–2.47)	0.293
Biclonal gammopathy	0.67 (0.16–2.88)	0.593	1.34 (0.67–2.67)	0.402
Clinical features				
Hemoglobin <100 g/L	1.06 (0.24–4.57)	0.940	1.25 (0.63–2.46)	0.523
Platelets <100 × 10^9^/L	**2.60 (1.13–5.98)**	**0.024**	1.43 (0.74–2.76)	0.290
Total WBC > 10 × 10^9^/L	1.51 (0.42–3.07)	0.795	1.04 (0.62–1.75)	0.876
Elevated LDH	1.65 (0.67–4.07)	0.281	**3.59 (1.85–6.97)**	**<0.001**
Elevated β_2_‐microglobulin	1.41 (0.32–6.19)	0.652	1.09 (0.15–8.18)	0.931
Splenomegaly	1.25 (0.50–3.13)	0.629	1.11 (0.49–2.49)	0.803
Cytogenetic abnormalities				
Presence of del(17p)	1.76 (0.55–5.65)	0.342	1.73 (0.43–7.01)	0.445
≥3 abnormalities	1.41 (0.49–4.08)	0.524	1.16 (0.28–4.92)	0.837
Immunophenotype				
CD56+	0.93 (0.34–2.58)	0.889	1.42 (0.54–3.71)	0.476
CD19 and/or CD20+	0.31 (0.09–1.10)	0.069	0.22 (0.05–1.00)	0.051

*Note*: The numbers marked in bold indicate they are statistically significant.

Abbreviations: HR, hazard ratio; ISS, international staging system; LDH, lactate dehydrogenase; R‐ISS, revised ISS; WBC, white blood cells.

^a^ Both were compared ISS or R‐ISS stage I.

^b^ Both were compared to IgG isotype.

### 3.4 Survival

The median follow‐up for the entire cohort was 92 months (95% CI, 63–121). Median PFS for pPCL and sPCL were 4.8 months (95% CI, 0.4–9.2) and 0.8 month (95% CI, 0.5–1.1) (*p* < 0.001; Figure 1A), respectively. Median OS for pPCL and sPCL were 18.3 months (95% CI, 0.0–39.0) and 1.2 months (95% CI, 0.9–1.5) (*p* < 0.001; Figure 1B), respectively. When considering survival from MM diagnosis to death, the median OS for sPCL was 30.2 months (95% CI, 24.1–36.2). The one‐month, one‐year, and three‐year survival rates were 97.0%, 68.9%, and 36.1% for pPCL and 57.6%, 7.6%, and 3.8% for sPCL. At the time of datalock (July 1st, 2022), 89 patients had died. The cause of death was available for 81 of them and all but one died of complications related to disease progression (mainly infections and bleeding).

As illustrated in Table 2, only one variable had a significant impact on pPCL and sPCL OS (respectively, thrombocytopenia and elevated lactate dehydrogenase [LDH]) and thus, multivariable analysis could not be performed for either subgroup. Although expression of CD19 and/or CD20 was not statistically significant, a trend toward improved survival was observed in patients expressing these markers in both pPCL and sPCL.

## 4 DISCUSSION

Poor prognosis is the hallmark of PCL. In a cohort described 35 years ago by Noel and Kyle,^33^ the median OS for pPCL and sPCL were, respectively, 6.8 and 1.3 months. With the advent of new therapeutic options and better supportive care, pPCL OS gradually increased, to reach approximately 20 months in the late 2000s.^32^, ^34^ Since then, pPCL survival has, however, stagnated and the latest studies (using the same diagnostic criteria) have reported similar OS.^26^, ^29^, ^31^, ^35^ Likewise, we did not observe a significant increase in pPCL OS between 2005–2012 and 2013–2020. For sPCL, our data also did not show improvement in survival over time. Moreover, almost half of the sPCL patients died within a month of transformation, similar the first cohort reported in 1974.^2^ As illustrated in Table S2, it is possible that the 2005–2012 and 2013–2020 cohorts had had different clinical characteristics, partly explaining the lack of improvement over time, but these data must be interpreted with great caution as the number of patients in several subgroups is very small. Hopefully, the advent of the latest generation of treatments regimens (such as those including anti‐CD38 monoclonal antibodies, bispecific antibodies, or CAR‐T) will have a greater impact on PCL survival than chemotherapy alone, but this remains to be determined since very few of our patients were exposed to these protocols and data from other research groups are scarce.^36^, ^37^, ^38^

Due its low incidence, the majority of data regarding PCL treatments comes from retrospective studies, limiting the ability to draw definitive conclusions. Nevertheless, one finding that is consistent across studies is improved survival in patients who undergo SCT.^17^, ^23^, ^24^, ^25^, ^26^, ^39^, ^40^ In our cohort, this type of treatment was also associated with better outcomes for pPCL patients. It is likely that patients who underwent ASCT or tandem ASCT‐alloSCT were fitter than patients treated with chemotherapy alone, but, interestingly, the median age at diagnosis and the proportion of patients with renal failure were similar in both groups. Regarding the optimal SCT approach (i.e., single ASCT vs. tandem ASCT vs. tandem ASCT‐alloSCT), different studies have reported conflicting data^15^, ^41^, ^42^ and thus, practice varies across institutions. Although it would have been interesting to assess the impact on survival of each SCT approach, the small size of our cohort prohibited this type of analysis.

While the majority of research groups have focused on therapeutic options, few studies have been dedicated to the clinical characteristics of PCL, particularly sPCL. Partly because of its very dismal prognosis, sPCL has been presumed to be the consequence of the long‐term selection of an aggressive clone in heavily treated MM. Our data suggests, however, that sPCL arises from an already high‐risk MM. Firstly, the OS from MM diagnosis to death was only 30.2 months, which is substantially shorter than high‐risk (defined as R‐ISS III) MM in modern cohorts (40–60 months^43^, ^44^). A second marker of poor prognosis noted in our MM population was the high rate of relapse within 12 months of SCT. Early relapse, an independent risk factor of short survival,^45^, ^46^, ^47^ usually occurs in 15%–20% of MM patients^46^, ^47^, ^48^ but it was observed in almost 50% of our cohort treated with SCT. Lastly, the proportion of patients with multiple cytogenetic abnormalities, another marker of poor prognosis,^49^, ^50^, ^51^ was notably higher in our cohort (36% of patients had ≥2 cytogenetic abnormalities) than in other studies (10%–14%^49^, ^51^).

Very few variables had a significant impact on OS in our cohort, which is likely attributable, at least in part, to the small size of various subgroups. Nevertheless, an intriguing finding of this study was the trend toward improved survival seen in patients expressing CD19 and/or CD20. In MM, CD20 expression strongly correlates with the t(11;14) translocation^52^, ^53^ and in a recent report by Cazaubiel et al.,^14^ this translocation was associated an improved OS in pPCL (39.2 vs. 17.8 months). Moreover, it has been demonstrated, in MM, that both CD20 positivity and t(11;14) are associated with a more lymphoplasmacytoid morphology.^52^, ^54^, ^55^, ^56^ Hypothetically, these markers may be associated with a propensity for plasma cells to circulate in the blood without being highly dedifferentiated, therefore explaining why these patients tend to have better outcomes despite high peripheral plasma cells counts. In our cohort, only a few patients were tested for the t(11;14) translocation and thus we could not assess whether CD19 and/or CD20 expression was only a reflection of this translocation or if it was a separate factor for improved survival. Furthermore, these results should be interpreted with caution as flow cytometry was only performed in approximately half of our patients.

There are a few limitations to this study, the first being its retrospective nature and the incomplete data inherent in this type of research. For example, it would have been interesting to assess clonal selection in sPCL between MM diagnosis and sPCL diagnosis. Unfortunately, very few patients had data available at both time points and thus, this type of analysis was not possible. The other main limitation is the use of the 2013 IMWG criteria instead of those of 2021. Because the research protocol was elaborated and submitted before the IMWG criteria update of December 2021,^4^ the patients in this study were selected based on previous criteria. As mentioned earlier, PCL criteria have been expanded following studies demonstrating that outcomes are similar for patients with ≥5% and ≥ 20% circulating plasma cells.^5^, ^6^ Consequently, we believe that our results remain of interest. Finally, despite being one of the largest cohorts of PCL reported, our population remains fairly small. Some analyzes may have been underpowered and should be repeated in a larger cohort.

## 5 CONCLUSION

In summary, our data suggest that leukemic transformation in sPCL occurs in already high‐risk MM. We also confirm the very bleak prognosis of both pPCL and sPCL. While SCT seems to be of benefit for eligible patients, we report no improvement in OS in recent years, highlighting the urgent need for basic research focusing on PCL in order to develop better treatment options.

## AUTHOR CONTRIBUTIONS

**Camille Tessier:** Data curation (lead); formal analysis (lead); project administration (lead); writing – original draft (lead). **Richard LeBlanc:** Data curation (equal); supervision (equal). **Jean Roy:** Data curation (equal); supervision (equal). **Sabrina Trudel:** Data curation (equal). **Julie Côté:** Data curation (equal). **Marc Lalancette:** Data curation (equal). **Jean‐Samuel Boudreault:** Data curation (equal); supervision (equal). **Émilie Lemieux‐Blanchard:** Data curation (equal). **Rayan Kaedbey:** Data curation (equal). **Michel Pavic:** Conceptualization (lead); data curation (equal); funding acquisition (lead); investigation (lead); methodology (lead); resources (lead); supervision (lead).

## FUNDING INFORMATION

This work was supported by the Université de Sherbrooke, via institutional funding.

## CONFLICT OF INTEREST STATEMENT

The authors declare no conflicts of interest relevant to the content of this article.

## ETHICS STATEMENT

The study protocol was reviewed and approved by the *Comité d'Éthique de la Recherche* (Research Ethics Boards) of the CHUM, the CHUQ, the CHUS, the Charles‐Le Moyne Hospital, the Jewish General Hospital, the Maisonneuve‐Rosemont Hospital and the Sacré‐Cœur de Montréal Hospital (multicentric approval number: MP‐31‐2022‐452).

## CONSENT

Because of the retrospective nature of the study, patient consent was waived by the Research Ethics Board of the CHUS.

## Supporting information


Data S1:

## Data Availability

The data that support the findings of this study are available from the corresponding author, upon request.
