# Supplementary material for: Poor outcome despite modern treatments: A retrospective study of 99 patients with primary and secondary plasma cell leukemia
Source: Cancer Med. 2024 Sep 3;13(17):e70192. doi: 10.1002/cam4.70192 (PMC11369989; doi:10.1002/cam4.70192)
Supplement: Supplementary file 1 — Data S1: [file CAM4-13-e70192-s001.docx]

**Supplemental Table I.** Therapeutic regimens used for first and second line for multiple myeloma (MM), secondary plasma cell leukemia (sPCL) and primary plasma cell leukemia (pPCL). The numbers in parentheses represent patients for whom the corresponding protocol was used as an induction regimen for stem cell transplant.

|  |  | **MM** | | **sPCL** | | **pPCL** | |
| --- | --- | --- | --- | --- | --- | --- | --- |
| **Treatment regimens** | **Abbreviation** | **1^st^ line** | **2^nd^ line** | **1^st^ line** | **2^nd^ line** | **1^st^ line** | **2^nd^ line** |
| Bortezomib, dexamethasone | Vel-Dex | 12 (7) | 10 | 4 | 1 | 5 (1) | 2 (1) |
| Bortezomib, doxorubicin, dexamethasone | PAD | 9 (7) | 3 (1) | 4 |  | 6 (5) | 1 |
| Bortezomib, lenalidomide, dexamethasone | VRd | 3 (1) |  | 1 |  |  | 1 |
| Bortezomib, melphalan, prednisone | VMP | 6 | 1 |  |  | 2 | 1 |
| Bortezomib, thalidomide, dexamethasone | VTD |  |  | 1 |  |  |  |
| Carfilzomib, lenalidomide, dexamethasone | KRd |  |  | 2 | 1 |  | 1 |
| Cyclophosphamide, bortezomib, dexamethasone | CyBorD | 12 (7) | 3 (1) | 2 | 1 | 11 (5) | 1 (1) |
| Daratumumab, lenalidomide, dexamethasone | DRd |  | 1 | 1 | 1 |  | 2 |
| Dexamethasone, (thalidomide), cisplatin, doxorubicin, cyclophosphamide, etoposide | D(T)-PACE |  |  | 1 |  |  | 1 |
| Lenalidomide, dexamethasone | Rev-Dex | 1 | 14 | 5 | 2 |  | 9 (1) |
| Melphalan, prednisone | MP | 9 (1) | 5 |  |  | 1 | 1 |
| Melphalan, prednisone, thalidomide | MPT | 2 | 1 |  |  |  |  |
| PAD alternating with CyBorD | PAD / VCD |  |  |  |  | 6 |  |
| Pomalidomide, cyclophosphamide, dexamethasone | Pom-Cyclo-Dex |  |  | 2 |  |  |  |
| Thalidomide, dexamethasone | Thal-Dex | 7 (2) | 3 | 4 |  | 2 (1) | 1 (1) |
| Clinical trial | - | 1 | 1 |  |  |  | 1 |
| Other treatment | - |  |  | 6 | 2 |  | 2 |

**Supplemental Table II.** Baseline clinical characteristics, by year of diagnosis, of patients with primary and secondary plasma cell leukemia (PCL).

| **PARAMETERS** | **Primary PCL** | | **Secondary PCL** | |
| --- | --- | --- | --- | --- |
|  | **2005-2012 (n = 10)** | **2013-2020 (n = 23)** | **2005-2012 (n = 29)** | **2013-2020 (n = 37)** |
| Age at diagnosis, years, median (range) | 59.4 (45.6 - 82.2) | 59.6 (40.7 - 86.3) | 65.2 (37.8 - 79.5) | 63.1 (41.4 - 85.3) |
| Male sex, n (%) | 6 (60.0) | 13 (56.5) | 14 (48.3) | 18 (48.6) |
| Plasma cells, median (range)  Circulating (%)  Circulating (absolute)  Medullary (%) | 25 (20 - 81)  3.3 (1.1 - 72.8)  95 (50 - 100) | 39 (11 - 80)  7.9 (1.8 - 62.6)  90 (50 - 100) | 29 (20 - 66)  1.9 (0.3 - 17.9)  75 (35 - 90) | 30 (17 - 76)  2.5 (0.3 - 81.5)  82 (14 - 100) |
| ISS stage, n (%)  I  II  III | 1/8 (12.5)  2/8 (25.0)  5/8 (62.5) | 2/19 (10.5)  5/19 (26.3)  12/19 (63.2) | 2/15 (13.3)  5/15 (33.3)  8/15 (53.3) | 2/16 (12.5)  1/16 (6.3)  13/16 (81.3) |
| R-ISS stage, n (%)  I  II  III | 0/5 (0)  3/5 (60.0)  2/5 (40.0) | 2/18 (11.1)  6/18 (33.3)  10/18 (55.6) | 0/10 (0)  6/10 (60.0)  4/10 (40.0) | 0/15 (0)  4/15 (26.7)  11/15 (73.3) |
| Paraprotein isotype, n (%)  IgG  IgA  IgM  Light chain only | 4 (40.0)  1 (10.0)  0 (0)  5 (50.0) | 6 (26.1)  2 (8.7)  1 (4.3)  14 (60.9) | 15 (51.7)  7 (24.1)  0 (0)  7 (24.1) | 14 (37.8)  11 (29.7)  0 (0)  12 (32.4) |
| Light chain isotype, n (%)  Kappa  Lambda | 5 (50.0)  5 (50.0) | 16 (69.6)  7 (30.4) | 14 (48.3)  15 (51.7) | 20 (54.1)  17 (45.9) |
| Biclonal gammopathy, n (%) | 3/10 (30.0) | 0/20 (0) | 3/26 (11.5) | 7/36 (19.4) |
| CRAB features, n (%)  Hypercalcemia  Renal failure  Hemoglobin < 100 g/L  Bone lesions | 5 (50.0)  5 (50.0)  8 (80.0)  5 (50.0) | 17 (73.9)  18 (78.3)  22 (95.7)  16/22 (72.7) | 11 (37.9)  16 (55.2)  25 (86.2)  9/15 (60.0) | 19 (51.4)  21 (56.8)  31 (83.8)  16/24 (66.7) |
| Other clinical features, n (%)  Platelets < 100 x 10^9^/L  Total WBC > 10 x 10^9^/L  Elevated LDH  Elevated β_2_-microglobulin  Positive Bence Jones  Immunoparesis  Splenomegaly | 5 (50.0)  6 (60.0)  5/9 (55.6)  7/8 (87.5)  6/7 (85.7)  9/9 (100.0)  3/9 (33.3) | 11 (47.8)  21 (91.3)  15/21 (71.4)  16/19 (84.2)  7/8 (87.5)  18/21 (85.7)  8/18 (44.4) | 25 (86.2)  6 (20.7)  16/28 (57.1)  15/15 (100.0)  10/14 (71.4)  23/23 (100.0)  4/8 (50.0) | 29 (78.4)  16 (43.2)  30/35 (85.7)  15/16 (93.8)  10/11 (90.9)  30/32 (93.8)  8/17 (47.1) |
| Cytogenetic abnormalities, n (%)  Normal FISH  Standard risk abnormalities  Trisomy  t(11;14)  High-risk abnormalities  t(4;14)  t(14;16)  Del17p  Gain 1q  Del1p  ≥ 2 abnormalities  ≥ 3 abnormalities | 2/5 (40.0)  2/5 (40.0)  0/1 (0)  0/5 (0)  0/3 (0)  0/4 (0)  n/a  n/a  1/5 (20.0)  0/5 (0) | 0/19 (0)  3/19 (15.8)  4/6 (66.7)  2/16 (12.5)  2/9 (22.2)  5/17 (29.4)  10/13 (76.9)  4/8 (50.0)  13/19 (68.4)  9/19 (47.4) | 0/5 (0)  1/5 (20.0)  2/3 (66.7)  2/3 (66.7)  n/a  0/3 (0)  0/1 (0)  0/1  3/5 (60.0)  2/5 (40.0) | 0/8 (0)  2/8 (25.0)  0/1 (0)  0/6 (0)  2/7 (28.6)  3/8 (37.5)  4/5 (80.0)  2/5 (40.0)  6/8 (75.0)  3/8 (37.5) |
| Immunophenotype, n (%)  CD56+  CD19 and/or CD20 + | 3/4 (75.0)  3/6 (50.0) | 6/17 (35.3)  4/16 (25.0) | 3/5 (60.0)  0/6 (0) | 9/16 (56.3)  3/17 (17.6) |

Abbreviations: *BM* bone marrow, *FISH* fluorescent in situ hybridization, *LDH* lactate dehydrogenase, *ISS* international staging system, *R-ISS* revised ISS, *WBC* white blood cells.
